# Video Synchronization With Bit-Rate Signals and Correntropy Function

**DOI:** 10.3390/s17092021

**Published:** 2017-09-04

**Authors:** Igor Pereira, Luiz F. Silveira, Luiz Gonçalves

**Affiliations:** Department of Computer Engineering and Automation, University of Rio Grande do Norte, Rio Grande do Norte 59078-970, Brazil

**Keywords:** correntropy, variable bit-rate, video synchronization

## Abstract

We propose an approach for the synchronization of video streams using correntropy. Essentially, the time offset is calculated on the basis of the instantaneous transfer rates of the video streams that are extracted in the form of a univariate signal known as variable bit-rate (VBR). The state-of-the-art approach uses a window segmentation strategy that is based on consensual zero-mean normalized cross-correlation (ZNCC). This strategy has an elevated computational complexity, making its application to synchronizing online data streaming difficult. Hence, our proposal uses a different window strategy that, together with the correntropy function, allows the synchronization to be performed for online applications. This provides equivalent synchronization scores with a rapid offset determination as the streams come into the system. The efficiency of our approach has been verified through experiments that demonstrate its viability with values that are as precise as those obtained by ZNCC. The proposed approach scored 81% in time reference classification against the equivalent 81% of the state-of-the-art approach, requiring much less computational power.

## 1. Introduction

The evolution of technology concerning sensor networks has allowed for applications with distributed live multimedia data used for visual measurements, such as automotive, surveillance and security measurements, and industrial control. Because multiple viewpoints of a scene add much more information to the measurement [1], a good measurement system requires a synchronization method between distributed cameras images to retrieve more accurate measures.

Two problems are often encountered when dealing with online synchronization across multiple video streams [2]. The first is related to the extraction of time descriptors and the algorithm implementation to calculate the time reference between them. The second concerns clock synchronization among heterogeneous devices, as video streams are time-dependent data and need to be acquired and reproduced with a constant time interval to preserve their meaning.

Clock drift may lead to two common buffer problems: buffer overflow and buffer underflow. Both are related to memory space availability and the initial buffer size (latency is the key). From the synchronization point of view, clock drift may result in a variable time reference between video streams.

Although a few synchronization methods require clock synchronization to ensure low latency and guaranteed Quality-of-Service, clock synchronization is not necessary to achieve video synchronization. In fact, because of the precision of today’s crystal oscillators combined with a good amount of available memory space at the receiver device, a non-synchronized multimedia system may take days to experience its first buffer error. However, from a practical viewpoint, clock synchronization is indeed necessary to provide guaranteed and high-accuracy measures. Therefore, we describe a few related works regarding clock synchronization in sensor networks.

Many of the video synchronization methods described in the literature use geometric correspondences or illumination-based analysis to extract good time descriptors. Although these methods present advantages in specific conditions, many of them rely on a large amount of processing power to analyze image data. This is a problem for nodes with limited processing power, which are common in sensor networks.

To solve these problems, we propose an online video synchronization method for distributed video cameras in sensor networks that is based on differential entropy analysis of encoded streams carrying variable bit-rate (VBR) data. The main advantage of this method is its low computational complexity in the extraction of a time descriptor, mostly because the instantaneous bit-rate measure is a property of an encoded stream and can be extracted without decoding. This is also one good reason that facilitates its use for the online synchronization of multiple video streams. Here we define online synchronization as a method for the synchronization of real-time signals being transmitted over sensor networks.

In order to find a temporal reference between video streams, we investigate the use of a generalized correlation function named “correntropy”. Correntropy is a similarity measure that includes higher-order statistical moments of the analyzed data, and is thus more efficient when dealing with non-linear and non-Gaussian signals.

In this paper, we discuss the correntropy time-lag analysis and the timing classification algorithm that we have developed. Section 1.1 and Section 1.2 present the related work concerning both the clock and video synchronization methods. Section 2 presents the background theory necessary to understand the proposed synchronization method. Section 3 discusses the timing reference classification algorithm we have developed. Section 4 presents the experimental results of the proposed synchronization method, and the overall conclusions are presented in Section 5.

### 1.1. Clock Synchronization

From a practical point of view, high-accuracy vision sensors often require clock synchronization. There are many industrial vision cameras equipped with dedicated coaxial clock synchronization input/output ports, in which a master device sends trigger signals to control the image acquisition of slaves’ devices. The main drawback of this approach lies in the limitations yielded by synchronization cables. In fact, long cables can degrade synchronization performance, while small cables constrain the spatial configuration of vision sensors. An alternative solution is to use a wired standard bus, such as IEEE1394 [3] or Ethernet [4], instead of the classical coaxial synchronization cables. Despite the flexibility provided by these systems, they still require physical connections and are unsuitable for wireless vision sensor networks.

An alternative to wired clock synchronization is to employ wireless communication protocols for synchronization in sensor network fields. However, most of the synchronization protocols depend heavily on the media access time, which is nondeterministic for wireless networks [5]. A few strategies were developed to avoid the nondeterminism problem. Synchronization methods such as the Timing-sync Protocol for Sensor Networks (TPSN) [6] and the Flooding Time Synchronization Protocol (FTSP) [7] suppress the non-determinism of the media access by time stamping the sent messages at the media access control (MAC) layer. However, they require special MAC implementations. Another method developed for time synchronization of wireless sensor networks is based on firefly-inspired algorithms [8,9]. Despite their accuracy and fast convergence [8], firefly-inspired algorithms are not compatible with most commercially available wireless sensors networks chips [9]; very few groups have used firefly-inspired synchronization. Some groups have adopted firefly-inspired synchronization on special platforms such as wire [10,11], ultra-wideband (UWB) [12] and custom radio [13] platforms. It is also possible to attach an external radio, global positioning system or optical device for the clock reference signal at the cost of additional equipment.

Lei Hou et al. developed a frame synchronization approach for high-speed vision cameras [14]. The idea of their approach is to use the incident light from an intensity-modulated illumination source as the reference signal to control a phase-locked loop (PLL) algorithm. The PLL algorithm generates a time reference signal to control image acquisition. Each camera synchronizes its time reference signal according to a single modulated illumination source in order to achieve clock synchronization between them.

### 1.2. Video Synchronization

Regarding video synchronization, some methods proposed in the literature are based on geometric correspondences of multiple images [15,16,17,18,19]. These methods are able to perform geometric calibration and synchronization at the same time. However, they rely on the assumption that there are a sufficient number of correspondences across images, which is not guaranteed for certain applications. Additionally, these methods experience an exponential decrease in performance when analyzing a considerable amount of video streams.

Another approach that uses space-time interest points is proposed by Laptev and Lindeberg [20] and is used for video synchronization [21]. In the method, a Harris corner algorithm detects the space-time points with scale-adapted techniques and selects these using a uniform search with a uniform sampling algorithm. This results in a distribution of space-time interest points, which represents a time feature descriptor of each video. Then, a correlation algorithm tries to estimate the temporal difference between these descriptors. However, this algorithm tends to fail for image sequences with foreground objects moving over background clutters [22].

The work of Ushizaki et al. [22] describes a method for image synchronization that uses appearance changes in the video sequences. In their method, the basic idea is to use the spatial integral over the image plane of temporal derivatives of brightness as a temporal feature of a video sequence. Then the normalized cross correlation function estimates a time delay between these temporal features. Although the scientific community devoted plenty of efforts to the image-based synchronization technique, the application of this technique in real-world situations suffers from a few innate limitations, such as prerequisite Light Emitting Diode (LED) auxiliary, arbitrarily tilting or stationary cameras, a specific texture of the background, or a restrictive motion of objects [14].

The Moving Picture Experts Group (MPEG) is working on a new standard to provide compact descriptors for video analysis [23]. The idea is to extract features from the keyframes of a video stream and append a set of descriptors for each feature. While this research is very interesting in the field of automatic content classification, it may not provide enough resolution to perform an accurate time reference calculation. Because the descriptors are based only on the keyframes, the group-of-pictures (GOP) structure plays an important role in the feature extraction. Therefore, small videos without any scene change may not provide a useful time descriptor for video synchronization.

Recent studies [24,25] present the properties and usage of a signal extracted from stream instantaneous bit-rate, which is the VBR, to retrieve timing information of recorded data from multiple cameras capturing the same scene. Al-Nuaimi et al. [25] developed an algorithm that is based on a measure called “consensus-based correlation”, or ConCor+, which uses a cross-correlation measure of pairwise VBR signals from multiple streams in conjunction with the random sample consensus algorithm (RANSAC) for timing classification.

The studies concerning the use of instantaneous bit-rate as a time descriptor [24,25] are the fundamental inspiration for devising our online synchronization method. The ConCor+ algorithm presented by Al-Nuaimi et al. [25] is our closest scientific reference with comparable results of those discussed in Section 4.

## 2. Background Theory

In order to better understand our proposal, it is necessary to understand the concepts regarding the VBR time descriptor extraction and the correntropy function usage for time-lag analysis.

### 2.1. The Use of VBR Signals

In general, video clips are composed by a sequence of images with high temporal correlation. Modern encoders describe the video information using differential encoding, which begins with the assumption that the decoder already has all the information necessary to decode the last frame. Hence, it is only necessary to transmit the changes from the last frame to the actual frame. This class of video encoders uses a motion-based technique to cope with the high temporal correlation of video data and to achieve lower rates. Therefore, the amount of data used to represent each frame is related to the amount of movement presented in the scene. A frame that is not distinguished from its neighbors presents vanishing conditional entropy; hence, its additional information becomes closer to zero [24].

**Figure 1:**
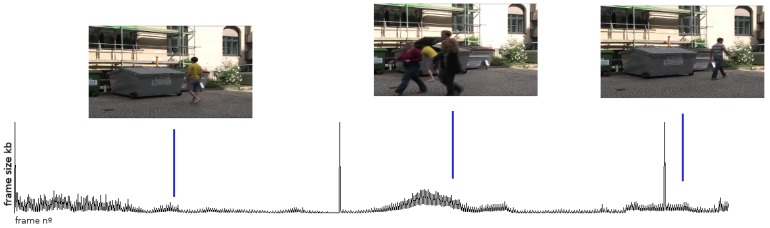
Variable bit-rate (VBR) signal from h264 codec.

An example of a VBR signal extracted from an h264 codec video stream is shown in Figure 1, wherein each point of the VBR signal represents the number of bytes per frame of the encoded stream.

A good time descriptor has the ability to distinguish between camera motion and scene motion. While the former does not carry any synchronization data and is irrelevant for the synchronization algorithm, the latter contains precious information closely related to synchrony. For the case of homogeneous motion patterns, which are related to camera movement, the motion-based prediction is highly efficient, resulting in a very low residual error. Homogeneous motion patterns are encoded with a very low data rate [24].

The main contribution to the bit-rate of a motion-based encoder is related to an object’s movement. Not only a moving object expose additional background that needs to be encoded and transmitted, motion vectors from object movement are also less regular and more difficult to compress [24].

Although the VBR signal does not require a huge amount of computational power to be extracted, given it is a property of an encoded stream, it is highly suitable for uncorrelated segments [24]. These uncorrelated segments yield false results from cross-correlation time estimation. A false estimation that happens as a result of an uncorrelated segment analysis is called an outlier. In another work found in the literature [25], the RANSAC is used to deal with the presence of outliers. The RANSAC algorithm estimates the parameters of a model in a two-step loop: the first step estimates the model parameters from a minimal sample set that is randomly selected; the second step checks which elements of the entire dataset are consistent with the model instantiated with the parameters estimated in the first step. After a predefined number of iterations, the RANSAC chooses the parameters of the model that had greater consistency among the entire dataset.

Despite the great results presented by the ConCor+ method [25], these were obtained from recorded video files and there is no mention of applications in real-time online video synchronization. We notice that the windowing strategy used by [25] is likely to be the main drawback that makes its application in real-time systems difficult. In this paper, we propose an algorithm for time reference classification of VBR signal pairs that is suitable for real-time applications using a different windowing strategy that is based on the correntropy function for time-lag analysis.

Correntropy is a similarity measure that can extract both second-order and higher-order statistical information from analyzed data [26]; this makes it suitable for the analysis of non-Gaussian and non-linear signals, which we believe is the case for VBR signals.

#### Influence of Encoding Parameters

The VBR signals are obtained with the x264, an open implementation of the H264/AVC encoder. Therefore, two important encoding parameters contribute to the success of this synchronization approach.

To ensure a VBR time descriptor representing more of the motion flow of the scene, a fixed quantizer must be used. This is set by configuring the Quantization Parameter (QP) to a user-specified quantization value.

Another parameter that needs to be set is related to the Group Of Picture (GOP) size and structure. The GOP size parameter represents the interval of frames in which an *i*-frame is sent. An *i*-frame contains all the information necessary to decode a frame. The encoder decides to send an *i*-frame according to a few rules. One rule is the detection of a scene change, which is not the case for the videos presented in the dataset; the other rule is a time interval measured in frames that force the encoder to start a new GOP. A point that should be noted is that a GOP is composed of an *i*-frame and following *b*-frames and *p*-frames. A *b*-frame uses information from the past and from the future to decode a specific frame, which can invert the order of the frames in the video stream. Therefore, the presence of *i*-frames and *b*-frames in the VBR signal can degrade the performance of the time reference classification algorithm. The *p*-frames are the frames that carry motion data as the time descriptor of an encoded stream. Hence, the *b*-frames are disabled and the GOP size is set to a specified value.

According to a detailed analysis performed by Florian [27], the synchronization error is reduced for QP values greater then 40 and a GOP size of 499. For a QP value greater then 40, the contribution of the motion vectors to the VBR signal is greater then the contribution of the quantization process. Additionally, the GOP structure may be present after the re-encoding process as a re-encoding artefact in the form of a spike pattern. In fact, the spikes are periodic with a period equivalent to the least-common multiple between the original GOP value $G_{0}$ and the new GOP value $G_{1}$. If the new GOP value $G_{1}$ is set to be a prime number, the spike period is set to $G_{0}*G_{1}$ samples. As a consequence of the increased period length, the spike disturbance takes on a more aperiodic character [27]. Hence, the chosen number for the GOP length was 499.

### 2.2. Correntropy Time-Lag Analysis

Correntropy is a generalization of the correlation measure that includes both second-order and higher-order statistical moments of the analyzed data [26]. Therefore, it works better than correlation when applied to non-Gaussian and nonlinear signals. This concept has been successfully applied in several engineering problems [28], such as in nonlinearity tests [29], in estimating a time delay from a signal pair [28], and in measuring respiratory and heart rates with a photoplethysmogram [30].

As defined by the principle in [26], a cross-correntropy measure taken between two random signals is given by

(1) $$\begin{array}{cc} v_{x,y;\sigma }\left(t_{1},t_{2}\right)= & E[k_{\sigma }\left(x_{t_{1}},y_{t_{2}}\right)]\\ = & \int \int k_{\sigma }\left(x_{t_{1}},y_{t_{2}}\right)p_{X,Y}\left(x_{t_{1}},y_{t_{2}}\right)dx_{t_{1}}dy_{t_{2}} \end{array}$$

where E[.] is the expectation operator, and $k_{\sigma }$, also called the “kernel function”, is any positive definite symmetric bivariate function. This work uses a Gaussian kernel, defined as

(2) $$k_{\sigma }\left(x,y\right)=\frac{1}{\sqrt{2\pi }\sigma }e^{\frac{-{\left(x-y\right)}^{2}}{2\sigma^{2}}}$$

where σ, the variance of the Gaussian function, is defined as the kernel size. The kernel size may be interpreted as the resolution, wherein the correntropy function measures the similarity in a space with high dimensionality.

Applying a Taylor series expansion to the correntropy measure of Equation (1), it can be rewritten as [26]

(3) $$V_{x,y;\sigma }\left(X_{t_{1}},Y_{t_{2}}\right)=\frac{1}{2\pi \sigma }\sum_{n=0}^{\infty }\frac{{\left(-1\right)}^{n}}{2^{n}\sigma^{2n}n!}E_{x,y}\left[{\left(X_{t_{1}}-Y_{t_{2}}\right)}^{2n}\right]$$

Note that the correntropy measure presents the sum of infinite moments of even order, and thus contains information from infinite statistical moments of its data. From Equation (3), it is possible to visualize how the kernel size parameter σ is related to the statistical moments in the correntropy measure. The second-order moments are dominant for large values of the kernel size, and the measurement becomes similar to that for correlation.

In practice, the joint probability density $p_{X,Y}\left(x_{t_{1}},y_{t_{2}}\right)$ is unknown, and an estimator should be used to extract it from the available data. Considering a finite amount of data ${\left(x_{n},y_{n}\right)}_{n=1}^{N}$ that is strictly stationary in all statistical moments, and using a probability density estimator based on the Parzen method, it is possible to define an estimator for cross-correntropy as [26]:

(4) $${\hat{U}}_{x,y;\sigma }\left(m\right)=\frac{1}{N-m+1}\sum_{n=m}^{N}k_{\sigma }\left(x_{n},y_{n-m}\right)$$

## 3. Proposed Method

**Figure 2:**
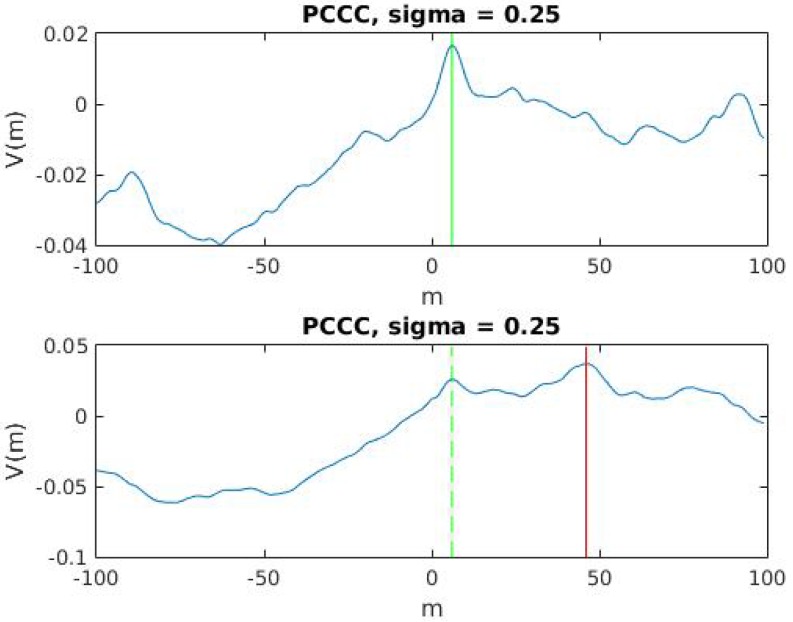
PCC of different windows. In upper row, the global maxima reveal the correct time offset. The lower row has its time offset revealed as a local maxima.

With the purpose of performing a simple and real-time time-lag analysis over a pair of VBR signals, these signals are segmented into the form of sequential windows of size *N*. For every window pair, *w*, a zero-mean normalization is applied to both signals and a Partial Cross-Correntropy estimator (PCC) is computed with Equation (4), by performing a sweep in the input value *m*, −2N/3≤m≤2N/3. It is presumed that the window size is at least 2/3 times higher than the expected drift value. Therefore, all windowed pairs should reveal the same global maximum as the correct time reference candidate. However, because of the presence of uncorrelated segments in VBR signals, not all of the PCC reveals the global maximum as the correct time reference. Some PCC reveal the correct time reference as a local maximum. An example of a PCC that yields an inlier and an outlier time reference candidate are shown in Figure 2.

**Figure 3:**
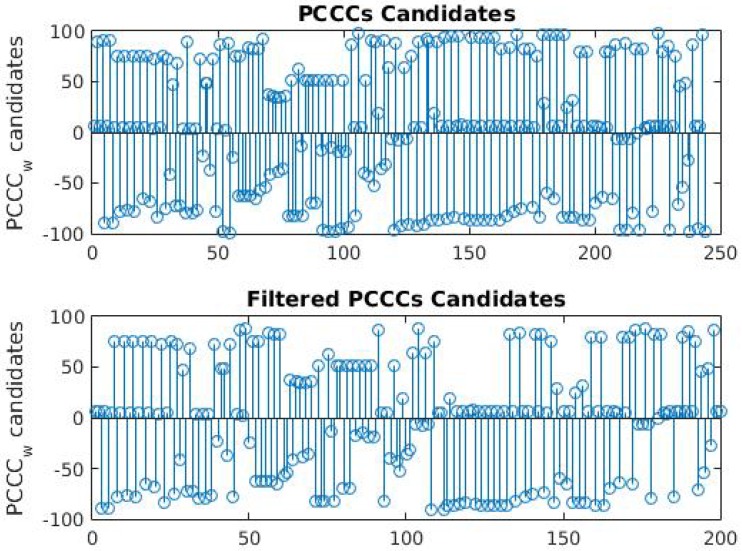
PCC candidates in upper row. PCC candidates after outlier filtering.

Additionally, uncorrelated window pairs can create untrustworthy peak values when *m* is near to 2N/3. This happens mainly because of the shape of the centralized cross-correntropy estimator. Because Equation (4) is applied in a finite sample set of size *N*, the number of analyzed samples N−m drops as *m* increases. This results in a poor estimation quality when *m* gets closer to *N*, and there is the possibility of a crescent function in the estimation results. This is also one of the reasons to sweep the *m* value from −2N/3 to 2N/3. Therefore, candidates extracted near to 2N/3 within a predefined range *f* are removed from the possible time reference set. Figure 3 demonstrate the results after an initial filtering of PCC candidates for f=10. In this example, the correct delay between the VBR signal pair is five samples.

Although in the example shown in Figure 3 the majority of candidates belong to the correct offset value, in many cases, a number of outliers and their distribution mask the correct time offset from the candidates. Thus, an efficient algorithm is necessary to deal with the presence of outliers and to perform a correct time offset classification.

### 3.1. Classification Algorithm

The algorithm proposed to perform a time offset classification is based on sample consensus, and the list of procedures is shown in Algorithm 1.

**Algorithm 1** Classification Algorithm
Strip signal $v_{1}$ and $v_{2}$ into overlapped segments $\left(v_{1,w}\right)$ and $\left(v_{2,w}\right)$ of size *N* and overlap ratio O.R..Compute PCCs for each segmented pair $\left(v_{1,w}\right)$ and $\left(v_{2,w}\right)$.Extract a Number of Candidates (N.C.) candidates’ offset (descent local maxima) from each PCC.For each candidate offset, evaluate the number of inliers. An inlier is any other candidate offset with its absolute distance within a predefined range ΔR.Choose the candidate offset with the highest number of inliers as the correct time offset between $v_{1}$ and $v_{2}$ signal pairs. The number of inliers divided by the total number of candidates gives the confidence level C.L.


The basic idea of the proposed algorithm to deal with the presence of outliers is to choose the most probable candidate offset within a predefined range. As can be seen in Figure 3, the correct time reference value forms a distribution density with a very small deviation while the outliers belong to a larger distribution, even if analyzed individually.

**Figure 4:**
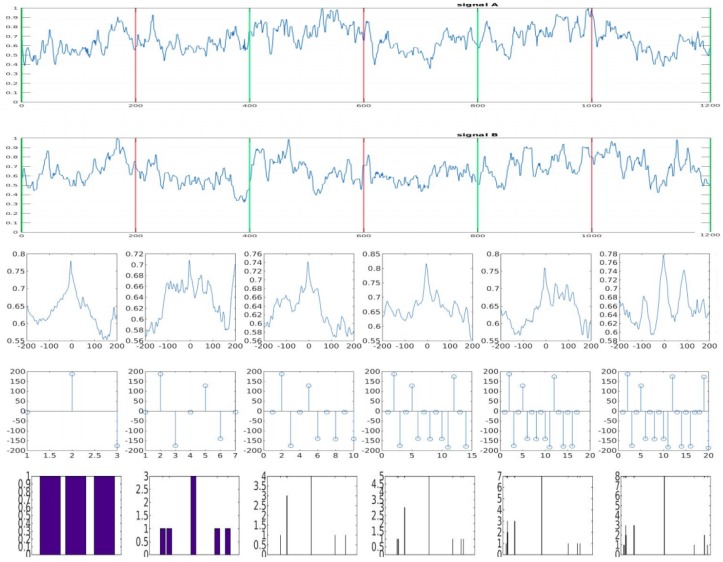
Signal flow of the proposed synchronization algorithm; N=400, O.R.=2, σ=0.20, N.C.=3, and ±ΔR=3.

Figure 4 demonstrates the signal flow of the proposed algorithm. The first and second lines show a VBR signal pair. Each of the other lines illustrate the signals obtained from each step of the algorithm. The VBR signals are chopped in sequential windows of size N=400 and overlap ratios O.R.=2; in Figure 4 the overlapped windows are between the vertical red lines. The third line shows the calculated PCC for each window. Then, a Number of Candidates (N.C.) in the form of local peak values are extracted for each PCC and are shown in the fourth line. The local peak values are cumulative, as they help increase the statistical precision and confidence. The last line shows the results of the classification algorithm in the form of a bar graph. Each bar in the plots from the fourth line represents the counting of each element within a predefined range. In the example of Figure 4, the parameters are configured as N=400, O.R.=2, σ=0.20, N.C.=3, and ±ΔR=3.

In the example of Figure 4, the proposed algorithm identified the correct time offset between the VBR signal pair at the second window analyzed. However, not all the cases converged as fast as that demonstrated in the example above. Although the proposed algorithm is very simple, there is a series of parameters that can be configured to maximize its performance. These parameters are described below and the experiments concerning them are described in the next section.

#### 3.1.1. Window Size *N* and Overlap Ratio O.R.

Considering the real-time requirements of the proposed synchronization model and the usage of the correntropy time-lag analysis function, the windowing strategy is chosen to allow the application of the correntropy time-lag estimator in parallel segment pairs of size *N*. These segments are extracted from each VBR signal sequentially with a degree of sample overlap O.R.

However, this introduces some drawbacks. The proposed method cannot extract a valid time reference from a pair of VBR signals if the absolute difference between them is greater than two-thirds of the window size. There are two factors that contribute to this behavior: the first is related to the *m* parameter of the cross-correntropy time-lag estimator, which assumes values limited between −2N/3 and 2N/3; the second concerns the parallel structure of the window analysis, as for both the VBR signals analyzed, the window pairs started and ended in the same sample.

We consider the following example, where the window size *N* is 900 samples. The maximum absolute offset value cannot exceed 600 samples. For the same window size N=900 and with an overlap ratio of O.R.=3, a new window starts at every 300 samples and extends for 900 samples. Additionally, for N=900 and O.R.=50, a new window starts at every 18 samples.

From a probabilistic viewpoint, there is an intrinsic relationship between the *N* and O.R. parameters. To increase the probability of a correct time reference classification, we should only select the windows without any uncorrelated segments. However, there is no simple way to perform this selection.

Our approach is to use sequential windows of size *N*, shifted by N/O.R. samples from the previous sample. For a small window size, the O.R. parameter contributes less to the probability of choosing a window free of uncorrelated segments, as the number of shifted samples N/O.R. is decreased when *N* is small. For bigger window sizes, the uncorrelated segments contribute less to an incorrect analysis if the majority of samples in the window are error-free. In this case, the O.R. parameter increases the number of properly analyzed windows, creating more trustworthy values.

For online synchronization systems, the window size and the overlapping ratio play an important role in the system response time to achieve synchronization. For each PCC, a time of (N/OR) samples is necessary to build a set of possible synchronization candidates. This means that the overlapping ratio parameter O.R. can decrease the system response time spent achieving synchronization for long window sizes.

Regarding computational complexity, this window strategy operates on a O(N) space for correntropy times the O.R. parameter, resulting in a O(N∗O.R.) space computational complexity.

#### 3.1.2. Inlier Range ±ΔR

In some cases, the majority of candidates extracted from the PCC of the whole signal may reveal the correct timing offset. However, the candidate distribution shows that the offset value lies within a range of values instead of at a single point. Therefore, the calculation of the time reference offset must consider values within a certain predefined range.

The idea behind the ±ΔR parameter is to separate the inlier candidates from the outliers by their distribution width. For now, this value is computed empirically, and an experiment regarding the ±ΔR parameter is described in the next Section.

#### 3.1.3. Confidence Level C.L. and Number of Candidates N.C.

The confidence level C.L. is an output parameter that measures the certainty in the result of a time-lag analysis. Furthermore, it is a measure of the precision of the classification, and it presents a reference value for considering whether the classification is reliable. The C.L. parameter is intrinsically related to the number of candidates extracted as the descent local maximum of each PCC${}_{w}$. The use of a descent local maximum to capture a possible number of candidates N.C. decreases the probability of one candidate to be the correct offset value by 1/N.C; in general, this decreases the confidence level of the time reference classifications.

## 4. Experimental Results

In this section, an analysis of the ConCor+ dataset [25] is discussed with the proposed synchronization algorithm. The dataset contained 39 scenes recorded with two to six unsynchronized cameras, wherein some were static cameras combined with handy recording over different viewpoints; hence, this resulted in 137 possible video combinations. The ground truth value of each combination was also obtained from the dataset [25]; this was verified by hand. Each of these combinations were encoded by the *libx264* encoder with default parameters, except for the following: QP=51, no b-frames, and GOP=499.

The dataset, however, contained a mixture of short and long signals that varied from 179 samples up to 10,091 samples. This introduced two problems: The first has already been discussed in Section 3.1.1 and is related to the maximum offset value that is suitable for analysis. The other problem is related to the amount of discarded combinations as a result of a window size that is greater then the signal length. Therefore, we developed a simple heuristic to associate the window size *N* with the signal length.

This section has the following organization: A simple heuristic for the adaptation of the window size and signal length is presented first. Then, individual experiments varying a few parameters of the algorithm are described, and after that, an overall synchronization score is presented. At the end of this section, a detailed comparison, including the computational complexity, with the state-of-the-art method [25] is performed.

### 4.1. Window Size Ratio N.R. Heuristic

Being *L* the minimum length of the signals that form a combination, and N.R., a constant scalar value; the resulting window size *N* is calculated for every combination by

(5) $$N=\frac{L}{N.R.}$$

Equation (5) ensures that the window size *N* is always shorter than the signal length *L*. Therefore N.R. indicates the amount of windows analyzed for each combination without taking the overlap ratio into consideration. Considering the overlap ratio parameter O.R., the number of windows analyzed for each combination is calculated by

(6) $$N.Ws=\frac{L}{NR*OR}$$

The results presented in this section, unless otherwise noted, are a function of the N.R. parameter. Therefore, the only restrictive rule concerns the maximum offset value that is suitable for analysis. For example, the RockI scene from the dataset [25] is composed of a signal *a* with a length equal to $L_{a}=1379$ samples and a signal *b* with $L_{b}=515$ samples. The pointed ground truth value of this combination is −999. In this case, our algorithm could not classify the combination, and to make for a fair analysis, combinations such as this were discarded from the dataset. Table 1 summarizes the number of combinations analyzed per N.R. value.

### 4.2. Experiment with Overlap Ratio O.R.

Table 2 demonstrates the effect of the overlap ratio parameter O.R. for different values of N.R. From a probabilistic viewpoint, the O.R. parameter increases the probability of selecting a window free of corrupted samples, as each new window is shifted by only a few samples from the last analyzed window. Therefore, the O.R. parameter increases the number of analyzed windows and, in general, provides better synchronization scores.

However, there is a balance between the number of uncorrupted windows and the number of corrupted windows, which is also increased. From an empirical analysis, we determined that O.R.=4 is a value that provides a good synchronization score with low computational complexity.

### 4.3. Experiment with Inlier Range ±ΔR

Table 3 demonstrates the minimum, average, and maximum rates of correctly synchronized video pairs as a function of the absolute inlier range ±ΔR. For this experiment, an offset value within an absolute range of 4 units from the proof value is considered correct.

From this empirical analysis, we determined that ±ΔR=3 is a good value to distinguish the inlier offset candidates from the outliers.

### 4.4. Experiment with Confidence Level C.L. and Number of Candidates N.C.

Table 4 shows the confidence level obtained as an average of all correctly synchronized video pairs by considering 69 possible combinations for N.R.=15. The results are presented as a function of the number of extracted candidates from each window N.C.

Indeed, there is a balance between a higher N.C., which will increase the probability that a candidate will be the correct offset value as an attempt to increase the correct candidate number of inliers, and a lower C.L. resulting from a number of outliers that are increased as well. We determined empirically that N.C.≤3 is a suitable value for obtaining a high synchronization score while maintaining a confidence level value that is very close to 50%.

### 4.5. Overall Dataset Score

**Figure 5:**
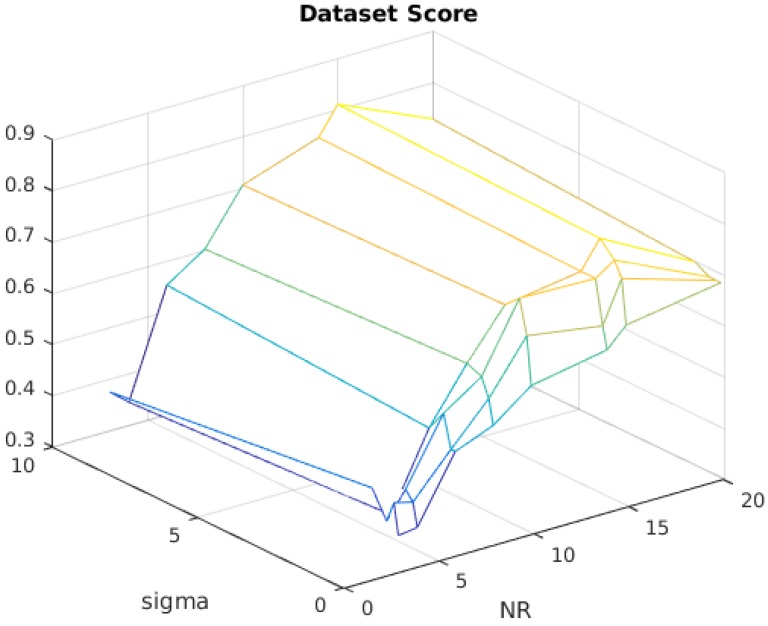
Dataset score; O.R.=4, ±ΔR=2, and N.C.=3.

As shown in Figure 5, the dataset analysis score of the time reference classification is presented as a function of σ per N.R. in the form of a surface. Synchronization attempts are only considered correct if they have an offset result within four frames of absolute distance from the ground truth.

Two important considerations can be drawn from Figure 5. The first concerns the parameter σ. For values of σ<1, the second-order statistics no longer contribute to the signal analysis. In fact, the width of the distribution projected by the Gaussian kernel function $K_{\sigma }\left(x,y\right)$ becomes thinner as the value of σ decreases. Therefore, the difference (x−y) from Equation (2) becomes more sensitive for sample values that are close to each other, making the parameter σ very related to the signal characteristics.

There are studies in the field of kernel density estimation that suggest a value for the parameter σ that is based on the signal behavior. In particular, for Gaussian kernels, the Silverman rule is a well-known method for estimating the kernel density [31]. However, it can yield incorrect results if the sample’s distribution is not close to a Gaussian distribution, which is not guaranteed for VBR signals.

Despite the fact that the correntropy function had a better score for σ≥1, when the correntropy function neared the correlation function, our classification algorithm with the correlation function at the core of its analysis did not yield better results. Table 5 shows a performance comparison between the correntropy and correlation functions, when one or the other was used at the core of the proposed classification algorithm.

As shown in Table 5, the correntropy measure has better results in the classification of VBR signal pairs. However, the difference between average and maximum rates reveals the need for heuristics that will better control the hard-coded parameters for maximum performance.

When analyzing the results from a varying N.R. perspective, we found that different combinations could yield correct results for different values of N.R. Varying the N.R. value according to the values in Table 1, we scored 72% with 84 correctly synchronized combinations of the total 117 combinations analyzed.

Although these results are equivalent with those of the state-of-the-art approach [25], a few considerations must be made when comparing both methods.

### 4.6. Detailed Comparison with State-of-the-Art Method [25]

A comparison of the proposed approach with the state-of-the-art algorithm described in [25] revealed some interesting results, which are discussed as follows.

The algorithm described in [25] presented very good results for determining time offsets between recorded video streams. However, there was no mention of its application in online synchronization, and for our consideration, their windowing strategy must be simplified in order to apply it to such a scenario.

The idea behind the ConCor+ algorithm [25], regarding its windowing strategy, is to split one of the signals into shorter segments $b_{i}$ of length *M*, and to cross-correlate each of them individually with the second signal *a*.

(7) $$b_{i}\left(t\right)=\left\{\begin{array}{c} b(t):(i-1)M\leq t<iM\\ 0:else \end{array}\right.$$

(8) $$c_{i}\left(\Delta t\right)=\sum_{t}a\left(t+\Delta t\right)b_{i}^{*}\left(t\right)$$

Then, the partial cross-correlation functions $c_{i}$ are combined in a RANSAC-motivated manner. While very effective, this windowing strategy has a computational complexity of $O\left(t*\Delta t*L_{b}/M\right)$, where $L_{b}$ is the length of the signal *b*. Therefore, the term $L_{b}/M$ represents the amount of segments *i* in which the signal *b* is divided. We note that t=M and $\Delta t=L_{a}$; hence, the ConCor+ windowing strategy computational complexity is

(9) $$O\left(L_{a}*L_{b}\right)$$

As state in Section 3.1.1, the computational complexity of our algorithm is O(N∗O.R.). When operating with the window size *N* related to the signal length *L* with the N.R. parameter, the resulting computational complexity is

(10) $$O\left(O.R.*L\right).$$

As demonstrated by Equations (9) and (10), our windowing strategy requires much less computational power, meaning that it can be used in the online synchronization of distributed cameras in sensor networks.

A closer look at the quantitative results revealed that our proposed approach scored a maximum of 81% in the offset determination against the same 81% scored from the ConCor+ [25] algorithm. Additionally, their average score obtained with hard-coded parameters was 57%, which was a little worse then our average result. However, the ConCor+ algorithm does not have the restrictions imposed by our windowing strategy; hence, it is able to analyze an entire dataset. By varying the N.R. parameter, we managed to analyze a bigger portion of the dataset, scoring 72% of 117 combinations. Still, these results can be improved with heuristics based on a probabilistic signal model to control the algorithm parameters.

## 5. Conclusions

In this paper, we investigate a technique regarding the synchronization of real-time multimedia streams that is based on the use of correntropy. The VBR signal is used as a time descriptor for offset determination, which, in conjunction to the correntropy function, leads to an algorithm with lower computational complexity when compared to the state-of-the-art algorithm [25]; thus it is more suitable for online video synchronization.

We proved that the correntropy function is more efficient in the time reference analysis over a pair of VBR signals than the correlation function. This means that the statistical moments of higher order play an important role in the rejection of outlier samples. We also demonstrate the results as a function of the parameter σ, which indicates the number of higher-order statistics included in the measure.

The use of the correntropy function in a simple windowing strategy allowed us to develop a simple classification algorithm that is able to increase the time reference statistics for every new window that is analyzed, increasing the confidence of the time reference classification. Therefore, our approach is more suitable for online synchronization of real-time streams in a distributed scenario.

### Future Work

As seen in the experimental results presented in this paper, the results of our proposed method are comparable to those of the state-of-the-art algorithm [25]. Our synchronization algorithm scored a maximum of 81% against the same 81% of the state-of-the-art algorithm. However, the difference between the average and maximum synchronization score of our algorithm indicates the need of a signal model that will better control the interrelations of the algorithm parameters for optimum performance.

Additionally, the classification algorithm can be improved in several manners. The use of the k-means clustering algorithm to eliminate outliers is another feature that we are currently developing. We believe that this will yield better synchronization scores and higher C.L. values. Our pending projects include the consolidation of the proposed time-lag classification algorithm with the creation of a signal model to control the algorithm parameters, as well as its implementation in the online synchronization of distributed systems with real-time requirements.

## Figures and Tables

**Table 1:** Number of combinations analyzed as a function of N.R. value.

**N.R.**	3	4	6	8	10	14	15	20
**No. of Combinations**	117	110	94	83	75	70	69	63

**Table 2:** Correctly synchronized video pairs as a function of O.R. and N.R.; σ=1, ±ΔR=4, and N.C.=3.

*O.R.*	*N.R.* = 4	*N.R.* = 10	*N.R.* = 15
**1**	31%	56%	62%
**2**	34%	68%	72%
**3**	43%	68%	75%
**4**	44%	74%	81%
**5**	42%	72%	79%
**6**	45%	77%	79%
**7**	45%	73%	79%

**Table 3:** Correctly synchronized video pairs as a function of ±ΔR; σ=1, O.R.=4, and N.C.=3.

Inlier Range	Min	Avg	Max
**2**	37%	60%	82%
**3**	36%	60%	82%
**4**	34%	59%	81%
**5**	33%	56%	78%

**Table 4:** Confidence level and synchronization score as a function of *N.C.*; σ=1, N.R.=15, O.R.=4, and ±ΔR=3.

*N.C.*	*C.L.*	Synchronization Score
**1**	85%	79%
**2**	46%	81%
**3**	45%	81%
**4**	44%	76%

**Table 5:** Comparison of synchronization scores among correntropy and correlation functions; O.R.=4, ±ΔR=4, and N.C.=3.

Function	Min	Avg	Max
Correntropy	34%	59%	81%
Correlation	14%	33%	62%
